# Innovative strategies for the management of long bone infection: a review of the Masquelet technique

**DOI:** 10.1186/s13037-015-0079-0

**Published:** 2015-10-14

**Authors:** Vivek Chadayammuri, Mark Hake, Cyril Mauffrey

**Affiliations:** University of Colorado School of Medicine, Aurora, CO USA; Department of Orthopaedic Surgery, University of Michigan, Ann Arbor, MI USA; Department of Orthopaedic Surgery, Denver Health Medical Center, University of Colorado, School of Medicine, 777 Bannock Street, Denver, CO 80204 USA

**Keywords:** Osteomyelitis, Posttraumatic, Defects, Diaphyseal, Reconstruction, Masquelet, Antibiotic, ORIF, Fixation

## Abstract

Post-traumatic long bone osteomyelitis (PTOM) is a relatively frequent occurrence in patients with severe open fractures and requires treatment to prevent limb-threatening complications. The Masquelet technique represents a length-independent, two-staged reconstruction that involves the induction of a periosteal membrane and use of an antibiotic-impregnated cement spacer for the treatment of segmental bone loss that result from bone infection. In this review, we summarize recent developments regarding the diagnosis and treatment of long bone PTOM, with a special emphasis on the use of the Masquelet technique for reconstruction of wide diaphyseal defects.

## Introduction

Osteomyelitis, or infection of the bone, represents a complex and challenging clinical entity in the field of orthopedics. In 1951, Gallie et al reported a case of recurring osteomyelitis following a period of 80 years since onset of initial infection. The patient was a 90-year-old woman with a Brodie’s abscess localized to the distal femur. Given a largely asymptomatic presentation throughout the patient’s lifetime, diagnosis and treatment was exceedingly delayed. This case is but one of many that illustrates the complex nature of osteomyelitis [1].

The focus of this review will be on long bone posttraumatic osteomyelitis (PTOM), defined as infection of the bone in conjunction with recent fracture or traumatic insult. Long bone PTOM is a relatively frequent occurrence and may be involved in as many as 10 % of all open fractures and 1 % of all closed fractures [2]. Several etiological factors have been previously described, including direct inoculation at time of injury, macro- or microvascular damage, surgical contamination, host immunodeficiency, and/or postoperative wound contamination [3–5]. Barring early diagnosis and adequate treatment, long bone PTOM may result in fracture nonunion, sepsis, and diffuse tissue devitalization underlying a requirement for limb amputation [4].

## Diagnostic evaluation of long bone PTOM

The clinical diagnosis of long bone PTOM is challenging, in large measure, owing to the non-specific nature of its initial presentation. In addition to findings of localized pain, long bone PTOM classically presents with signs and symptoms of infection including low-grade fever, erythema, edema, and/or draining sinus tracts [5, 6]. In pediatric patients, presentation may also include systemic manifestations such as fever, chills, and night sweats [5]. Clinical examination should reveal localized tenderness to palpation overlying an aspect of bone with prior or current fracture.

Diagnostic work-up of long bone PTOM traditionally involves a combination of imaging, tissue culture, and laboratory studies [7]. Acute inflammatory markers such as erythrocyte sedimentation rate (ESR), C-reactive protein (CRP), and leukocyte count (WBC) have low specificity for diagnosis, particularly in the setting of a profound systemic inflammatory response (e.g. rheumatoid arthritis, Crohn’s disease, systemic lupus erythematosus, etc.). Therefore, trending levels of acute inflammatory markers is more appropriately reserved for monitoring infection status following initiation of treatment [5, 6]. The ‘gold standard’ for the diagnosis of PTOM is a bone culture obtained in the operating room; however, this should always be complemented by histopathological analysis to reduce the incidence of false-positives [7, 8]. Currently ongoing clinical studies are investigating newer technologies such as polymerase chain reaction (PCR) and fluorescence in situ hybridization (FISH) that may improve diagnostic sensitivity; however, additional research is required to inform the feasibility and validity of such modalities [8, 9].

Following positive findings on culture and pathology suggestive of long bone PTOM, preoperative work-up must include an imaging series consisting of anteroposterior (AP) and lateral radiography, MRI, and/or CT. A multimodal imaging approach is employed to surmount the independent limitations of each imaging modality. In patients with long bone PTOM, AP and lateral radiographs traditionally demonstrate regional osteopenia, periosteal reaction (“Codman’s triangle”), and sequestrum (segments of necrotic bone with interspersed viable granulation tissue). However, sensitivity of plain radiography is quite poor during the initial two weeks of infection [5]. Computed tomography (CT) may enable earlier detection of infection through visualization of devitalized cortical bone, sequestrum, and/or involucrum (periosteal bone formation) on multiplanar reconstructions but demonstrates poor capacity to delineate soft-tissue involvement [10]**.** Magnetic resonance imaging (MRI) enables excellent visualization of soft-tissue pathology in as early as 3–5 days following initial onset of long bone PTOM [10] and is therefore considered the ‘gold standard’ for confirmation of osteomyelitis infection via imaging studies. Exudate, edema, or sequestrum appear as hypodense lesions on T1-weighted MRI images and short-tau inversion recovery (STIR) sequences, while surrounding granulation tissue appears as a low-intensity signal on T1-weighted images and high-intensity signal on STIR sequences or T2-weighted imaging [10]. The downside of MRI use is the artifact in the presence of hardware.

The classification of long bone PTOM is done according duration (Waldvogel et al) or disease stage (Cierny-Mader et al) and may facilitate treatment planning [11]. In the Walvogel classification, osteomyelitis is classified on the basis of being hematogenous, contiguous, or chronic in nature. In the Cierny-Mader classification, osteomyelitis is categorized by anatomic location into Stage 1 (medullary), Stage 2 (superficial), Stage 3 (localized), and Stage 4 (diffuse). This scheme also incorporates consideration of the host’s health status, divided into local factors (chronic lymphedema, venous stasis, or arteritis) or systemic factors (malnutrition, renal failure, diabetes mellitus, and immunodeficiency status). Both classification systems can be useful for informing diagnosis and treatment of long bone PTOM; however, a critical shortcoming is that anatomical staging within these classification schemes is often confounded by the presence of orthopedic hardware that generates artifact interference on imaging. In addition, no classification schemes incorporate the location of the infection on long bones (intra articular, metaphyseal or diaphyseal), which in our view is an important consideration for the treatment plan.

## Pre-operative optimization of the patient

The management of long bone PTOM is complex and challenging. Therefore, patients must be prepared for a long course of multiple surgeries and counseled on the risks for postoperative complications that include nonunion, hardware failure and infection recurrence. Moreover, initial stages of preoperative planning must involve correction of modifiable co-morbidities and/or risk factors that portend poor postoperative wound healing. Indeed, a study by Brinkler et al showed that 31 of 37 patients (83 %) with fracture nonunion had one or more underlying metabolic or endocrine abnormalities such as vitamin D deficiency, calcium imbalances, central hypogonadism, thyroid disorders, and parathyroid hormone disorders. Eight of these patients (25 %) achieved bony union in an average of 7.6 months (range, 3 to 12 months) following treatment of their metabolic or endocrine abnormalities without further operative treatment [12]. In addition to nutritional and metabolic testing, patients with long bone PTOM should also be evaluated for poor glycemic control (in diabetic patients), tobacco or illicit drug use, malnutrition, and vascular insufficiency of the affected limb [13]. Low socioeconomic status (SES) is also a prognostic indicator of worse treatment outcome [14], a fact that should not deter the provision of care but rather alert the treating surgeon to the increased potential for postoperative complications.

### Surgical treatment options

In severe cases of long bone PTOM, debridement of the infected tissue results in extended diaphyseal loss of bone that cannot be adequately managed by conventional methods of reconstruction. In particular, conventional methods often fail to satisfy at least one of the following goals of therapy:

I. Control of the local infection with radical debridement and antibiotic therapy
II. Fracture stabilization when instability occurs due to debridement or nonunion
III. Provision of adequate soft-tissue coverage to ensure wound healing [2, 5]

Radical debridement may further precipitate widening of the osseous defect in cases where segmental bone defects exceed 5 cm in size [15]. Conventional techniques such as vascularized fibula autograft and Iliazarov bone transport also yield poor long-term outcomes, often due to graft resorption and revascularization by creeping substitution [16, 17]. These treatment options are technically demanding and typically are not performed without specialized training. Bone regeneration may also be impeded secondary to inadequate vascularization or soft-tissue coverage [18]. Finally, insufficient delivery of concentrated antibiotic therapy to the site of infection may result in high rates of disease recurrence.

The Masquelet technique represents a two-staged reconstructive procedure that overcomes several of the shortcomings in the treatment of osteomyelitis defects, particularly those located to the long bone and associated with infected and/or non-viable soft tissue [19]. First developed in the late 1970’s by AC Masquelet but only recently popularized, the chief advantages of this strategy include control of the local infection with radical debridement, placement of a polymethylmethacrylate (PMMA) cement spacer for maintenance of dead space, and induction of a periosteal membrane that protects against graft resorption. Furthermore, the Masquelet technique is length-independent and is therefore a viable option for the treatment of larger osseous defects. A detailed description of the Masquelet technique with ‘Tips & Tricks’ and an illustrative case example are provided in the following sections.

## Two-staged reconstruction of extended diaphyseal bone defects using the masequelet technique

### Stage 1

#### A. Radical debridement

The Masquelet technique is performed in two stages. In the setting of an unstable long bone with infected and/or non-viable soft tissue, the first stage involves radical debridement of all infected or non-viable bone and interposed fibrous tissue. Given that devitalized tissue serves as a nidus for recurrent infection and predisposes to increased risk of postoperative complications such as delayed union, nonunion, and vascular thrombosis, reconstruction of the bone defect (conducted in Stage 2) is only possible once complete eradication of infected and non-viable tissues has been achieved. The margins of debridement should extend until viable bony edges are encountered, determined intraoperatively using the “paprika sign” (punctate bleeding upon drilling with a 2.5 mm drill bit). Once the margins of debridement have been determined, an osteotome can be utilized to perform a corticotomy in order to prevent destruction of healthy surrounding tissues.

#### B. Limb stabilization

Following debridement, stabilization must be achieved to maintain length, alignment and rotation prior to insertion of the cement spacer. Options include unilateral or ring external fixation, plate osteosynthesis, or IM nailing. The choice of stabilization is based on the location of the defect. For bone loss in the mid-diaphysis, an IM nail offers stable fixation that allows early weight bearing. A narrow diameter nail is used and coated with antibiotic-impregnated cement. For defects close to an articular surface, external fixation is preferred. Ring-fixators offer stable fixation and the ability to modify the bony alignment postoperatively. When placing external fixation, care must be taken to keep pins away from the site of definitive fixation so that the external fixator can be left in place until healing is achieved (during Stage 2 of the procedure) [18].

In our experience, the preparation of an antibiotic IM interlocking carbon-fiber nail can be achieved using a simple and reproducible technique [20]. Plastic tubing such as chest tubes, D&C tubing and D&E tubing are used as a mold. The tubing is cut to a length such that the proximal threaded portion of the nail is left free of cement. The inner portion of the tube is coated with sterile mineral oil to facilitate extraction of the nail. One end of the tubing is then clamped with a Kocher while the other end is loaded with the viscous cement-antibiotic preparation using a cement gun. The authors use 3 g of Vancomycin powder combined with 40 g of Palacos-R (Zimmer, Warsaw, Indiana) PMMA cement. When polymicrobial of gram-negative infection is suspected or demonstrated by bone cultures, 3.6 g of Tobramycin can be added to the mix. An extra 10 to 20 cc of monomer should be used to obtain injectable cement, and mixing should be performed under a vacuum to improve antibiotic elution profile [21]. An IM nail is then inserted centrally into the chest tube to produce a wide cement mantle with a consistent thickness. The entire construct is then placed into a cool sterile saline bath during the exothermic polymerization process to prevent melting of the inner layer of the plastic tube and facilitate removal of the nail [22]. The authors recommend allowing the interlocking holes to be covered with cement. Once hardened, the chest tube is cut longitudinally and peeled off of the cement-coated nail. The distal end of the cement mantle can be contoured with a rasp to allow for easier insertion. The intramedullary canal is then prepared in a standard fashion for nail insertion. The cement-coated nail is often wider than standard nails so aggressive reaming may be necessary prior to placement. If there is concern for proximal or distal spread of the infection in the canal, a Reamer-Irrigator-Aspirator (RIA) system may be used. The IM nail is then inserted and statically locked under fluoroscopic guidance, with proper alignment confirmed by plain radiography.

#### C. Placement of the antibiotic-impregnated cement spacer

The next step following bony stabilization is the fashioning of a cement spacer, traditionally composed of polymethyl methacrylate (PMMA) cement, to fill the segmental bone defect. A cement spacer is preferable over other modalities of local antibiotic delivery primarily because it assumes the conjoint functions of inhibiting fibrous tissue ingrowth and maintaining dead space volume until time of reconstruction [15]. For optimal outcome, the cement spacer should fill the intramedullary canal and edges of surrounding viable bone [18]. It is important to use a spacer to fill the entire defect as opposed to antibiotic cement beads. A membrane will form around the cement, which will be filled with bone graft during the second stage of the procedure. Beads leave an irregular membrane that is less than ideal for containment of the graft. Furthermore, the authors recommend irrigating with cool saline during the exothermic polymerization process to prevent local tissue necrosis. The cement spacer may also be premixed with antibiotic to enable localized delivery of higher concentrations than would be feasible with systemic therapy. This practice also provides a convenient and controlled dosing scheme that relinquishes issues related to poor patient compliance [23, 24]. 

The appropriate choice of antibiotic therapy is predicated on the results of culture and pathology testing performed on direct wound and bone samples. Additionally, the chosen antibiotic must be thermostable to the exothermic polymerization (solidification) process of the PMMA cement. Aminoglycosides (gentamicin, tobramycin) and vancomycin represent good options given their thermostability, broad-spectrum of activity, high rates of elution, and relatively low incidence of anaphylactic reactions [25, 26]. In an in vitro study by Chang et al, the longest duration of antibiotic-elution from PMMA cement spacers was observed with gentamicin as compared to vancomycin, teicoplanin, ceftazidime, imipenem, piperacillin, or tobramycin [27]. In this study, gentamicin also demonstrated excellent coverage against methicillin-sensitive *Staphylococcus aureus*, coagulase negative *Staphylococci, Pseudomonas aeruginosa* and *Escherichia coli* species. In another study performed in 15 mongrel dogs, Adams et al compared the elution characteristics of PMMA spacers loaded with cefazolin (Ancef; 4.5 g/40 g cement powder), ciprofloxacin (Cipro; 6 g/40 g powder), clindamycin (Cleocin; 6 g/40 g powder), ticarcillin (Ticar; 12 g/40 g powder), tobramycin (Nebcin; 9.8 g/40 g powder), and vancomycin (Vancocin; 4 g/40 g powder). Clindamycin proved to have the best elution profile in seromas, granulation tissue, and bone. High concentrations of tobramycin were observed accumulating in bone and granulation tissue, compared to high concentrations of vancomycin occurring in bone alone [28]. In an in vitro study by Penner et al, the elution of tobramycin and vancomycin from Palacos-R cement was found to persist over the course of 9 weeks [29]. This study also demonstrated that the dual administration of these two antibiotics increased antibiotic elution by 68 % for tobramycin and 103 % for vancomycin as compared to the use of each antibiotic independently. Finally, Wahlig et al. determined local concentrations of gentamycin to be as high as 80 mg/mL after 4 days post-implantation in a series of 41 patients treated with gentamycin impregnated PMMA beads [30].

Of note, the largest permissible ratio is 8 g of antibiotic per 40 g of cement**;** higher doses of antibiotic may impede cement molding [31]. During placement of the cement block it is critical to irrigate the spacer with cold saline during its polymerization phase as the raised temperature may cause skin burn.

#### D. Soft-tissue coverage and wound healing

In the final phase of stage 1, there must be closure of the wound without tension. This may require treatments ranging from wet-to-dry dressings to a flap procedure to provide adequate soft-tissue coverage. As a guiding principle, the least technically demanding strategy that enables successful soft-tissue coverage should be chosen. For acute injuries, free-flaps are preferred over rotational muscle flaps, as the latter can potentially increase destruction of viable surrounding tissue [14]. In a prospective study of 11 patients undergoing reconstruction of diaphyseal defects averaging 10.5 cm (range, 5 to 18 cm), 6 patients (54.5 %) required soft-tissue repair by flaps (3 free flaps, 3 pedicled muscle flaps). At 24 month follow-up, all flaps were viable and there was no recurrence of infection [19]. In Masquelet’s first report of the technique, 28 of 35 patients (80 %) undergoing reconstruction for long bone segmental defects (range, 5 to 24 cm) required soft-tissue repair procedures (14 free-flap, 14 pedicled muscle flap). Thirty-one patients (89 %) were able to resume unprotected weight-bearing at a mean of 8.5 months (range, 6 to 17 months). Four patients (11 %) sustained late stress fractures and required further cast immobilization to achieve complete healing. There were no cases of infection recurrence, which the authors attribute to aggressive initial debridement [19].

Wound vacuum-assisted closure (VAC) can also be used to promote tissue granulation, reduce tension required for wound closure, and minimize postoperative complications [32]. This is believed to occur secondary to a variety of mechanisms: (1) increased endothelial proliferation and angiogenesis, (2) increased tensile force that promotes tissue granulation and accelerated wound closure, and (3) presence of an airtight negative-pressure seal that reduces interstitial edema [33]. While data on clinical outcomes following use of wound VAC therapy in patients with osteomyelitis remains limited, one retrospective study found that patients with osteomyelitis treated by wound VAC experienced significantly lower rates of infection recurrence and required less flap procedures relative to patients treated by conventional wound management strategies. Debridement time and type was similar between the two groups [34]. In a separate study of 20 pigs with open fractures treated by antibiotic-impregnated PMMA beads containing vancomycin and tobramycin, concentrations of locally eluted antibiotics were unaltered by the application of wound VAC therapy [35].

On a final note, a randomized control trial performed by Bouachour et al demonstrated potential benefit with treatment by hyperbaric oxygen therapy (HBO) in patients suffering severe crush injuries of the limbs [36]. The study involved 36 patients with severe (grade III) crush injuries who were randomly allocated into HBO or placebo treatment groups within 24 h of surgical reconstruction. Compared to the placebo group, patients receiving HBO had significantly lower requirements for additional surgical procedures (flaps, grafts, vascular surgery, or amputation); moreover, complete wound healing was achieved in 87.5 % of patients receiving HBO compared to only 30 % of patients receiving placebo. Hence, augmentation of surgical reconstruction of severe crush injuries with HBO may improve clinical outcome; however, additional studies are required to inform this treatment strategy.

Following completion of stage 1 of the Masquelet procedure, weight-bearing is determined based upon the stability of the defect size, location and implant. Patients with small and medium diaphyseal defects treated by IM nailing can bear weight as tolerated. The patient is then placed on a prolonged systemic antibiotic regimen for a period of 6-8 weeks. This is done in order to allow adequate time for a number of processes to occur: (1) epithelialization of free or pedicled muscle flaps in order to prevent surgical site contamination by bacterial skin flora, (2) revascularization of marginally viable tissue surrounding the bony defect, (3) formation of the self-induced periosteal membrane, and (4) treatment of any residual infection by systemic and/or local antibiotics [19, 23, 37].

### Stage 2

#### E. Clearance of infection

Complete eradication of infection is a prerequisite to reconstruction of bone defects due to osteomyelitis (Stage 2 of the Masquelet technique). Trending inflammatory markers before and after completion of systemic antibiotics can help confirm clearance. If there remains any doubt as to the presence of residual infection, then tissue specimens at the site of the segmental defect can be harvested for culture and pathology [18]. Sending samples for pathology is critical due to the high rate of false-negative culture results [8]. Levels of acute inflammatory markers, including CRP and ESR, should be normal in patients lacking comorbidities [15]. In the second stage of reconstruction, the cement spacer is carefully removed and the resulting cavity is filled with morcelized autogenous corticocancellous bone graft.

#### F. Removal of the cement spacer and permanent fixation of the fracture

A single longitudinal incision is made centrally through the self-induced periosteal membrane. The cement spacer should be removed in one piece or a few small pieces created with a saw or osteotome. Particular care must be taken to avoid iatrogenic injury to the induced periosteal membrane so that it remains a self-contained compartment. The ends of the resected bone margins should be freshened with a drill bit or rasp to remove sclerotic bone and facilitate bone graft integration. The medullary canal should also be debrided to enable communicate with the graft. Definitive fixation can be revised at this point if necessary.

#### H. Harvest of autogenous bone graft using the Reamer/Irrigator/Aspirator (RIA) system

The hollowed periosteal cavity is best filled with morcelized autogenous bone graft. A synergistic effect between the bone graft and the induced membrane promotes increased bone formation, angiogenesis, and consolidation of the bony defect through stimulating the release of growth factors such as VEGF, TGF-beta 1, and BMP-2 [19, 38–40].

Bone graft can be harvested from a number of locations, including the iliac crest, proximal tibia and calcaneus. Use of the RIA system from the femur is preferred and portends less morbidity than iliac crest bone graft harvesting [41–43]. Furthermore, RIA aspirate has been shown to contain osteoprogenitor cells and tissue growth-factors (BMP-2, FGF-2, IGF-1, and TGF-β) that may accelerate bone repair [44, 45]. In a prospective study of 10 subjects, Sagi et al determined that aspirate obtained from medullary canal of the femur via RIA contained significantly higher levels of osteoinductive compounds compared to conventional iliac crest bone graft harvests [45]. A cadaveric study by Kovar et al further determined a significantly greater quantity of bone graft to be harvestable from the medullary canal of the femur compared to the tibia using RIA reaming [41].

While preparing for graft harvest using the RIA system, a few important considerations must be kept in mind. The reaming head size should not exceed the canal diameter at the isthmus of the femur (as determined on AP and lateral radiography) by more than 2 mm [42]. Reaming should be performed under fluoroscopic guidance using an alternating motion of advancing and withdrawing at a slow enough pace to allow proper irrigation and aspiration [42]. Once reaming is complete, aspiration should be turned off to reduce intraoperative blood loss.

Harvested bone graft should be loosely packed to bridge the osseous defect. It is important to avoid tight packing of bone graft when bridging the defect, as this can precipitate necrosis of the graft due to impaired angiogenesis. Large defects may require additional augmentation of autogenous bone graft with allograft or demineralized bone substitute at a ratio less than or equal to 1:3 (autograft to allograft) to achieve sufficient graft volume or strength [18, 19, 46]. Autogenous bone graft may also be enhanced with synthetic bone morphogenetic protein (BMP) [47, 48], bisphosphonates [18], or hydroxyapatite [18, 48]; however, the clinical utility of such synthetic derivatives remains controversial. Indeed, Masquelet et al observed increased autograft resorption in patients receiving additional local injections of recombinant BMP-7 [19].

#### I. Postoperative course

Following surgery, the patient is encouraged to resume immediate weight-bearing as tolerated. Early weight-bearing stimulates secondary bone healing (callus formation) and may help to reverse long-standing physical and psychological disability. The patient should be scheduled for routine follow-up postoperatively to evaluate for fracture alignment, osseous consolidation, and functionality.

With careful planning and execution, reconstruction of long bone osteomyelitis defects using Masquelet technique can yield excellent long-term clinical outcomes. In a case series of 25 patients presenting with 27 segmental bone loss nonunions averaging 5.8 cm in size (range, 1 to 25 cm), 24 cases (90 %) demonstrated full clinical and radiographic healing within 1 year following reconstruction with Masquelet technique. No postoperative complications, including infection recurrence, were reported [49].

The technique described here to treat long bone osteomyelitis is a feasible option for most orthopaedic surgeons. The materials required (PMMA cement, antibiotic powder, D&C tubing and mineral oil) are readily available at most centers. Carbon fiber products are becoming more popular and readily available, although a standard metallic nail can be substituted if necessary. Compared to alternative techniques such as bone transport and vascularized fibular grafting, the Masquelet technique is often technically easier and can produce good outcomes in a majority of patients.

## Case description

**Fig. 1:**
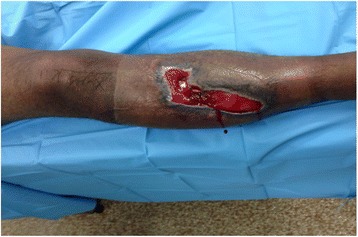
Preoperative clinical photograph demonstrating an anterior wound with exposed bone

As an illustrative case example, we present a 33-year-old male who sustained an open (Gustilo IIIB) diaphyseal fracture of the right tibia following an occupational forklift accident. Initial treatment was performed at an outside facility and included multiple rounds of debridement followed by open reduction and internal fixation (ORIF) of a buttery fragment along the medial tibia with locking plate and IM nailing. The patient was placed on wound VAC therapy for three months and subsequently developed a chronic draining wound over the anterior tibia with concomitant osteomyelitis (Fig. 1), prompting referral to our Level I trauma center.

Upon initial presentation to our institution, the patient was noted to have a foul-smelling wound with inflammatory hypergranulation surrounding an open 3 × 2 cm bony defect. The patient reported a deep, throbbing pain localized to the right anterior tibia with an intensity of 7/10 at rest. Past medical and family history were not significant for any metabolic, endocrine, or chronic inflammatory conditions. The patient reported smoking one-half pack of cigarettes daily for the past 10 years.

**Fig. 2:**
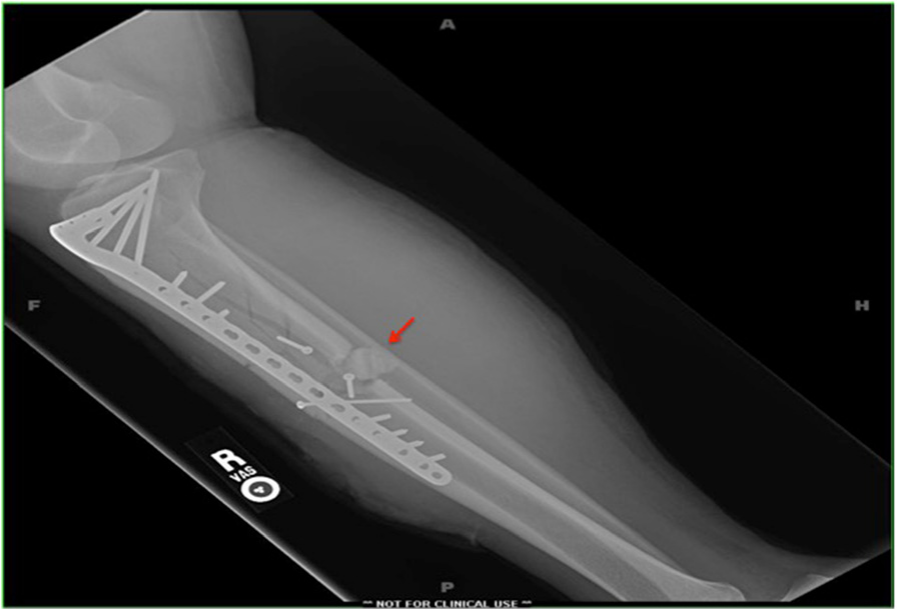
Preoperative lateral radiograph demonstrating a mid-diaphyseal comminuted fracture of the right tibia and sequestrum (red arrow)

On physical examination, the patient had decreased sensation to light touch over the cutaneous distribution of the superficial peroneal nerve and had drastically reduced strength of ankle dorsiflexion (1/5) in the right leg. Diagnostic imaging with anteroposterior (AP) and lateral radiographs revealed a mid-diaphyseal comminuted fracture nonunion of the right tibia with overlying soft-tissue swelling (Fig. 2). Direct bone samples were obtained for culture and pathology, which demonstrated a polymicrobial infection comprised of Methicillin-resistant *Staphylococcus aureus* (MRSA) and *Streptococcus anginosus*. The patient was started on a 6-week course of Vancomycin (15 mg/kg IV q8 hr) and Metronidazole (500 mg PO q8 hr).

Given the patient’s chronically infected nonunion and extensive necrosis of surrounding soft-tissue, a two-staged reconstruction using the Masquelet technique was performed. First, plate and screws were removed. Intraoperatively, absence of pinpoint bleeding was noted along an 11 cm segment of devitalized bone. Under fluoroscopic guidance, a Monotube multiplanar external fixator (Stryker, Kalamazoo, Michigan) was placed. Tibial osteotomy was carried out using an oscillating saw to resect necrotic bone, followed by radical debridement of all nonviable surrounding tissue. A PMMA cement spacer with 3.6 gm tobramycin and 3 gm vancomycin was prepared and placed into the bone defect. Soft-tissue coverage was provided using a rotational soleus flap. Incisional wound VAC therapy was applied for 1 week to promote tissue granulation and accelerated wound closure. The patient received a 6-week course of Vancomycin and Flagyl after direct tissue cultures grew *MRSA* and *Streptococcus milleri*.

**Fig. 3:**
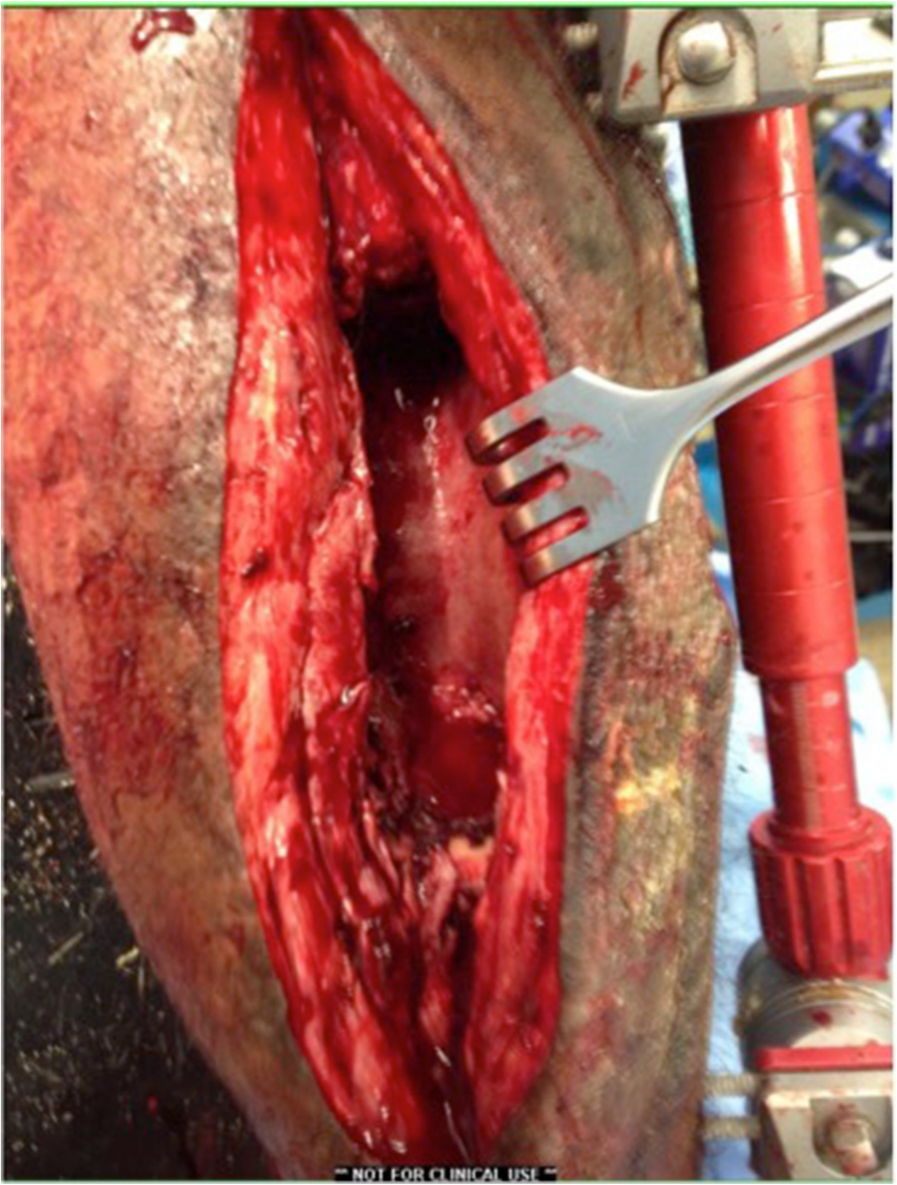
Intra-operative photograph of the self-induced periosteal membrane during the second stage of reconstruction following removal of the cement spacer. A cement coated antibiotic nail was placed to provide bone stability and allow early weight bearing

**Fig. 4:**
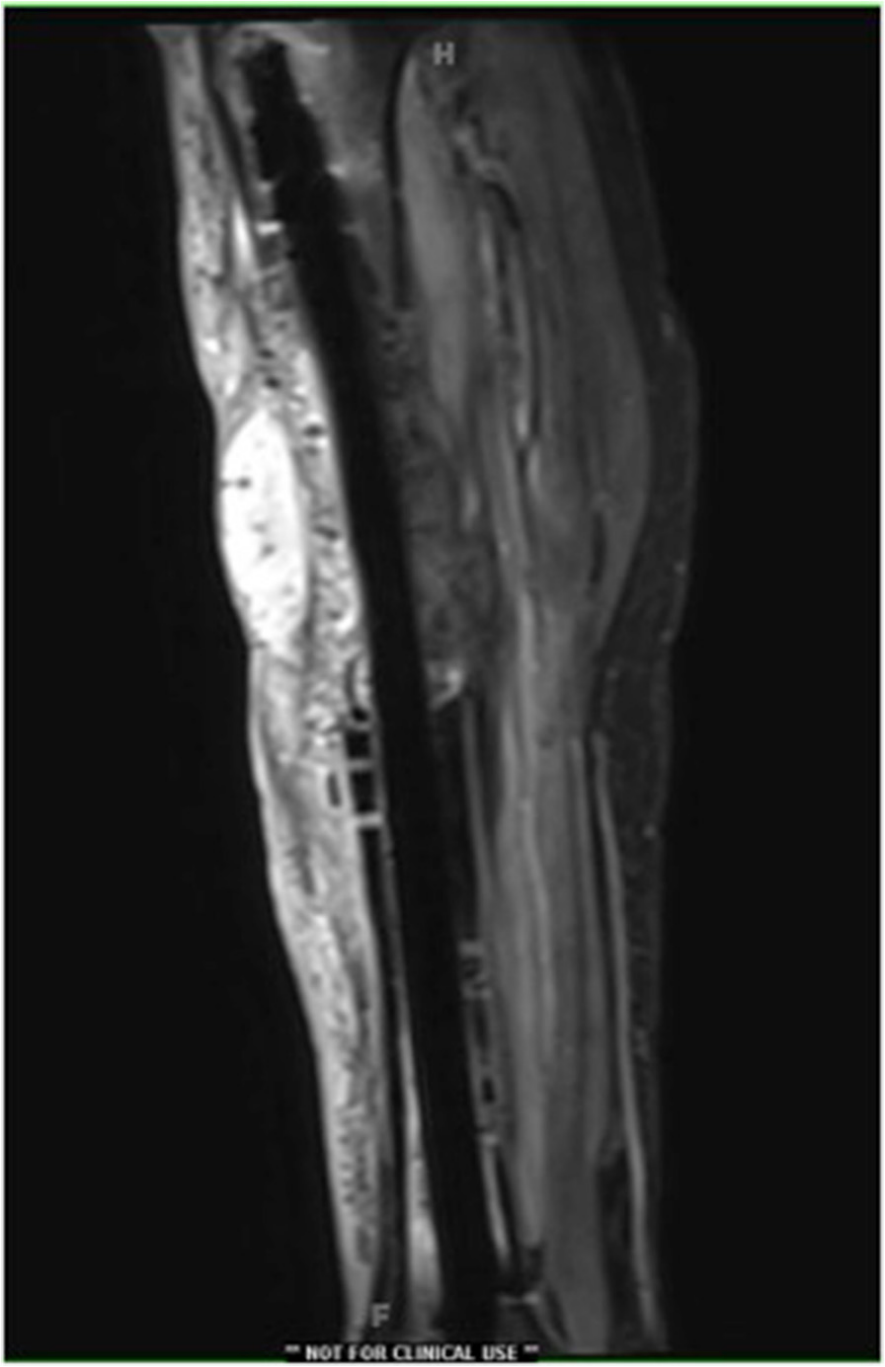
Postoperative MRI of the tibia following definitive fixation with radiolucent antibiotic-impregnated carbon-fiber IM nailing. Use of the carbon-fiber IM nail enables artifact-free MRI visualization

Eight weeks post-operatively, the patient returned to undergo the second stage of reconstruction. Intraoperative bone and tissue samples were culture negative. A significant self-induced periosteal envelope was visible overlying the previously placed cement spacer (Fig. 3). A central incision of the periosteal membrane was made in line with the tibia. The cement spacer was removed and pins of the external fixator were backed out until they were unicortical in nature. A 10-mm radiolucent carbon-fiber intramedulary nail (Carbo-Fix, Collierville, TN, USA) was coated with Palacos R cement (Warsaw, IN, USA) premixed with 3 g vancomycin and 3.6 g tobramycin. Under fluoroscopic guidance, the nail was positioned in an anterograde manner and locked with 2 proximal interlocking titanium screws (Fig. 4). The ipilateral femur was used for autograft harvest using RIA, which was loosely placed into the defect. The soft tissues, including the periosteal membrane, were closed in layers.

### Patient outcome

**Fig. 5:**
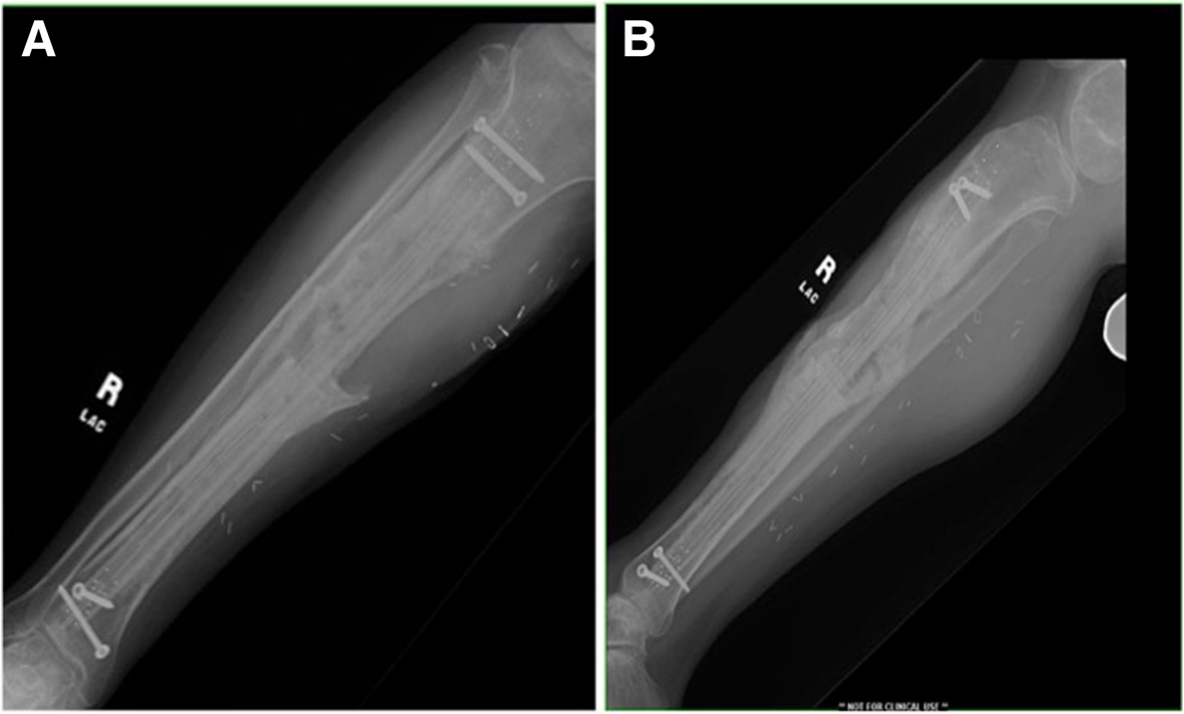
Standard AP (**a**) and lateral (**b**) plain radiograph taken at 5 months postoperative follow-up demonstrating improved bone healing without evidence of osteolysis, infection, or hardware migration

The patient was followed at 2, 8, 12, and 20 weeks postoperatively. The patient was able to resume full weight-bearing by 2 weeks postoperatively and was pain free at his 3 month follow-up visit. Radiographs at his 5 month visit showed consolidation of the defect without evidence of infection, osteolysis, or hardware failure (Fig. 5).

## Concluding remarks

- ✓ The Masquelet technique is a viable option for treatment of long bone PTOM. Primary advantages of this technique include its length independence, induction of a periosteal membrane that protects against graft resorption, and eradication of infection with an antibiotic-impregnated cement spacer that preserves dead space volume for delayed reconstruction.
- ✓ Radical debridement should extend to viable bone margins (as indicated by the paprika sign). Use of an osteotome to perform corticotomy helps prevent damage to healthy surrounding tissue.
- ✓ Following thorough debridement, an antibiotic-impregnated PMMA cement spacer is placed. Irrigation with cold saline during preparation of the antibiotic-cement mixture will help prevent skin burns.
- ✓ Stabilization during the first stage can be achieved with an external fixator, plate or IM nail.
- ✓ The cement spacer must be left in place for 6-8 weeks. Complete eradication of infection, confirmed by culture and pathology, is a prerequisite to the second stage of the procedure (reconstruction of the osseous defect).
- ✓ In the second stage, we favor definitive fixation using an antibiotic-coated carbon fiber IM nail. This allows for artifact-free visualization on MRI, which is important for monitoring treatment response.
- ✓ Autograft harvested using an RIA system should be loosely packed around the IM nail to permit angiogenesis. Bony margins should be freshened with a drill bit to facilitate graft integration.

## Consent

Obtained from patient.
